# Supplementary material for: Influence of sediment characteristics on the composition of soft-sediment intertidal communities in the northern Gulf of Mexico
Source: PeerJ. 2015 Jun 16;3:e1014. doi: 10.7717/peerj.1014 (PMC4476102; doi:10.7717/peerj.1014)
Supplement: Table S1 [file peerj-03-1014-s001.docx]

| **Site** | **Median Grain Size (μm)** | **Salinity (ppt)** | **TOC (%)** | **Sediment Grain Size CV** | **Shannon’s Diversity** | **Taxonomic Richness** |
| --- | --- | --- | --- | --- | --- | --- |
| **DIP** | 441.46 | 37 | 0.914 | 40.35 | 1.06 | 7 |
| **DIS** | 521.52 | 14 | 0.22 | 27.55 | 0.38 | 3 |
| **ELMS** | 178.48 | 8 | 0.59 | 30.64 | 0.14 | 5 |
| **ELMBB** | 171.91 | 55 | 1.98 | 25.02 | 0.31 | 4 |
| **WAVE** | 284.89 | 5 | 0.27 | 34.44 | 0.16 | 5 |
| **OS** | 116.37 | 8 | 5.1 | 100.36 | 0.59 | 10 |
| **CAM** | 126 | 12 | 1.91 | 22.72 | 0.16 | 3 |
